# Thermal performance of the Chagas disease vector, *Triatoma infestans*, under thermal variability

**DOI:** 10.1371/journal.pntd.0009148

**Published:** 2021-02-11

**Authors:** Sabrina Clavijo-Baquet, Grisel Cavieres, Avia González, Pedro E. Cattan, Francisco Bozinovic

**Affiliations:** 1 Laboratorio de Etología, Ecología y Evolución, Instituto de Investigaciones Biológicas Clemente Estable, Montevideo, Uruguay; 2 Departamento de Ecología, Center of Applied Ecology & Sustainability (CAPES), Facultad de Ciencias Biológicas, Pontificia Universidad Católica de Chile, Santiago, Chile; 3 Facultad de Ciencias Veterinarias y Pecuarias, Universidad de Chile, Santiago, Chile; Universidad de Buenos Aires, ARGENTINA

## Abstract

Vector-borne diseases (VBD) are particularly susceptible to climate change because most of the diseases’ vectors are ectotherms, which themselves are susceptible to thermal changes. The Chagas disease is one neglected tropical disease caused by the protozoan parasite, *Trypanosoma cruzi*. One of the main vectors of the Chagas disease in South America is *Triatoma infestans*, a species traditionally considered to be restricted to domestic or peridomestic habitats, but sylvatic foci have also been described along its distribution. The infestation of wild individuals, together with the projections of environmental changes due to global warming, urge the need to understand the relationship between temperature and the vector’s performance. Here, we evaluated the impact of temperature variability on the thermal response of *T*. *infestans*. We acclimated individuals to six thermal treatments for five weeks to then estimate their thermal performance curves (TPCs) by measuring the walking speed of the individuals. We found that the TPCs varied with thermal acclimation and body mass. Individuals acclimated to a low and variable ambient temperature (18°C ± 5°C) exhibited lower performances than those individuals acclimated to an optimal temperature (27°C ± 0°C); while those individuals acclimated to a low but constant temperature (18°C ± 0°C) did not differ in their maximal performance from those at an optimal temperature. Additionally, thermal variability (*i*.*e*., ± 5°C) at a high temperature (30°C) increased performance. These results evidenced the plastic response of *T*. *infestans* to thermal acclimation. This plastic response and the non-linear effect of thermal variability on the performance of *T*. *infestans* posit challenges when predicting changes in the vector’s distribution range under climate change.

## Author summary

The Chagas disease is transmitted by the infected feces of kissing bugs, such as *Triatoma infestans*, when it feeds on human blood. This species is an ectotherm and its physical activity level, behavior, and survival respond to environmental changes in temperature. Climate change predicts changes in environmental temperature and its variability. These changes may affect the distribution and survival of *T*. *infestans*, generating difficulties for eradication programs. Here, we evaluated the effects of temperature, and its variability on the performance of *T*. *infestans’* individuals maintained at or acclimated to six different temperatures, which capture the essence of climate change predictions regarding temperature (*i*.*e*., temperatures displaying changes in mean and in variance). We found that *T*. *infestans* exhibited a plastic response to thermal conditions, with individuals performing worse at a low mean temperature (18°C) with variance (± 5°C), while individuals acclimated to the same low temperature (18°C) without variance (± 0°C) performed as good as individuals acclimated to their optimal temperature (27°C). Nevertheless, thermal variation at a high mean temperature (30°C ± 5°C) increased performance. Thus, we concluded that the thermal variability affects the performance of this species, in a non-linear way. From our results, the difficulty to predict climate change effects on populations of *T*. *infestans* becomes evident, as well as the need to include putative thermal plasticity in future predictions.

## Introduction

The relationship between climatic variability and the increasing rate of the emergence of infectious diseases is currently one of the most important ecological problems [1–3]. Several infectious diseases have expanded their geographical range, or their incidence has incremented in areas where they were usually constrained [4], in some cases, in association with temperature increases. Moreover, some diseases have more recently reemerged in South America (*e*.*g*., Dengue fever and the Zika virus) in places where they had been gone for more than 100 years [5, 6]; or the diseases have expanded their range of occurrence (*e*.*g*., canine visceral leishmaniasis [7]). In this regard, vector-borne diseases (VBDs) are particularly susceptible to ongoing climate change since their incidence depends on the vector’s distribution, abundance, life-history traits, and vital rates; and most such disease-vectors are ectothermic arthropods [8]. Thus, their vital rates, life-history traits, and behavior are directly and strongly influenced by temperature [9]. However, it is puzzling how a vector’s distribution and the incidence of a disease will change with climate change [10]. Some authors suggest that the expansion of their distribution is more plausible [11], whereas some others point out that a shift in their distribution is more likely [8]. From this debate, the idea has emerged that in order to make proper predictions on how climate change will affect the occurrence of VBDs, the mechanisms dictating the relationship between temperature and vector-fitness must be fully understood [11]. One powerful and under-used tool to understand climate change effects on diseases is the basic reproductive number (R_0_) of a disease, which is the number of secondary cases that emerge from each single infected case [12]. For VBDs, the R_0_ is influenced, among other factors (*e*.*g*., the human vector ratio or the parasite’s extrinsic incubation period), by the vector’s survival rate [9, 13, 14]. Hence, vector control and eradication plans are the aim of health organizations to prevent VBDs’ transmission. Therefore, understanding how a vector’s survival is related to temperature changes when everything else remains constant, is the first proxy to be used in elucidating climate change effects on the occurrence of VBDs.

The relationship between temperature and an ectotherm’s life-history traits or fitness is non-linear [9, 15–18], positing difficulties to predict changes in vector diseases under scenarios of climate change, where extreme events and increments in temperature variability are projected [19]. In this sense, thermal performance curves (TPCs) are useful to understand the non-linear relationship between an individual’s fitness and changes in temperature, to then forecast effects of climate change on vector populations or distributions [20]. Numerous traits related to function capacity are usually used as performance measure to build TPCs, as for example survival, fecundity, development, or locomotion. In a typical TPC, the function measures changes rapidly and reversibly in response to temperature, increasing until a maximum performance (P_max_) is achieved at the optimal temperature (T_o_). After the temperature optimum, the performance decreases quickly [15, 18]. The points where performance reaches zero are denominated as the critical thermal minimum and maximum (CT_min_ and CT_max_, respectively) and they delimit the performance breath (T_br_). Critical thermal limits (or lower and upper thermal limits, LTL and UTL, respectively) are the temperatures where organisms are ecologically dead [21–24]: individuals cannot move, feed, reproduce, or perform any function for population maintenance.

Theoretical models predict that under thermal variability, the thermal niche will be narrower than that predicted under constant temperatures due to the reduction of lower and upper thermal limits [18]. Besides, it is also predicted that the P_max_ could be lower with an increase in temperature variability, whereas an increase in average temperature and in its variance might increase population variability, favoring for example pest outbreaks [18]. Along these lines, the study of performance of vectors and their response to acclimation at different temperature changes (*i*.*e*., in mean and in variance) are required to bring light to the debate of consequences of climate change and the emergence of diseases, such as VBDs.

The Chagas disease, also known as the American trypanosomiasis, is one neglected tropical disease [25], caused by the protozoan parasite, *Trypanosoma cruzi* (Trypanosomatidae). This VBD is mainly constrained to the Americas, where at least 6 million people are infected [26]. Just in Colombia, the medical costs related to Chagas are estimated to be about US$ 267 million, per year [27]. The most important vector of Chagas in southern South America is *Triatoma infestans*, a domestic triatomine, which usually lives in wall cracks of poorly-constructed homes, in rural areas [26]; and peri-domestic structures, such as chicken coops, pigsties, and barns [28]. This vector expanded its geographical distribution during the Spanish colonization of South America, from valleys in Bolivia and north Argentina to non-native areas in central Chile, some regions of Argentina, South Brazil, and Uruguay [29–32]. Nevertheless, after successful eradication programs, mostly in areas where the vector was introduced by human migrations, this vector established wild populations [33, 34]. Wild foci have been described in Chile [33, 34], Bolivia [35], and Argentina [36], with individuals living in natural micro-habits such as vegetation formations of bromeliads (*i*.*e*., *Puya spp*.) and stone-refuges, such as pircas [33, 37], and have probably been feeding on wild rodents [33, 35]. These wild foci posit challenges when trying to control diseases because they provide a refugium from which peridomestic habitats can be periodically re-colonized [35]. Therefore, the presence of sylvatic foci of *T*. *infestans*, even in non-native areas for *T*. *infestans*, together with the projections of global warming, urge the need to understand the relationship between temperature and performance in the main vector of the Chagas disease in southern South America.

Thermal tolerance in vectors of the Chagas disease has been previously studied through the estimation of the critical thermal minimum (CT_min_) and maximum (CT_max_) in *T*. *infestans* and in *Rhodnius prolixus*, the last a Central American Chagas’ vector [38]. The authors found that *T*. *infestans* exhibited a wider thermal tolerance than *R*. *prolixus*, with a range of 53°C between both thermal limits. They also found that the geographic distribution of *R*. *prolixus* and *T*. *infestans* was strongly associated with minimal winter temperatures, and for the case of *T*. *infestans*, a micro-habitat effect on its geographic distribution was also observed [38]. Regarding the thermal response of *T*. *infestans* to acclimation, this species showed thermal plasticity in CT_min_ in individuals acclimated for one week to four constant temperatures, from 14°C to 35°C. Nevertheless, its CT_max_ and its chill coma recovery did not show any acclimation response; and there is no study so far evaluating the response of *T*. *infestans* to temperature fluctuations in the environment, a fundamental feature of projected climatic changes [39]. Moreover, TPCs have not been estimated in this species despite their relevance to assess non-linear relationships between temperature and performance. Hence, we studied the thermal sensitivity of *T*. *infestans*, under six thermal treatments, with changes in mean temperature and temperature variability. Thermal sensitivity was studied through the estimation of TPCs.

## Methods

### Ethics statement

All the experiments using mice were conducted in agreement with the ethical standards and according to the local animal protection law. All experimental protocols were reviewed and approved by the Scientific Ethical Committee for Animal and Environment Care and the Scientific Ethical Committee for Research Safety of the Pontificia Universidad Católica de Chile (Protocol #160517015 for Biosecurity and Ethical protocols). These protocols are in accordance with the basic principles set forth in Chilean Law, 20, 380, on Animal Protection (2009), the European Directive 2010/63 / EU, and the Guide for the Care and Use of Experimental Animals (NRC, 8th Edition, 2010), documents to which this institution ascribes.

### Animal husbandry

A total of 133 third to fifth-instar nymphs of *Triatoma infestans*, from the colony of the Faculty of Medicine, of the Universidad de Chile were moved after feeding to the laboratory of Ecophysiology of Pontificia Universidad Católica de Chile. Individuals were then maintained individually in plastic vessels (3.8 cm x 6.8 cm) inside climatic chambers at controlled temperatures with a photoperiod of 12L:12D and 50% to 70% humidity. During the experimental period, the kissing bugs (*T*. *infestans*) were fed with sedated mice from the university’s animal house, despite that the individuals from the original colony were fed with avian blood. Thus, to avoid any confounding effect related to food type, the animals were maintained at the same temperature as in their original colony (*i*.*e*., at 27°C), with a photoperiod of 12D:12L, for one month, in order to acclimatize to the new food source, prior to being assigned to the respective experimental thermal treatments. During the entire experimental period, animals were fed in groups of five individuals per sedated mouse, to make sure that individuals were feeding *ad libitum*. Individuals were weighed in a BOECO (Model BAS 31 plus) before and after feeding to corroborate blood consumption. Sedation of mice was done with an intraperitoneal injection of 80 mg of Ketamanie and 10 mg Xilacine per kg. Sedated animals are a commonly used blood source to feed triatomines [40–42]. This procedure does not seem to affect *T*. *infestans* individuals, but rather protects them from putative defensive injury caused by the animals they are feeding on.

### Experimental design

Individuals were randomly assigned to one of following six thermal treatments that differed in their mean temperature and in its variance: 18°C ± 0°C (N = 17), 18°C ± 5°C (N = 24), 27°C ± 0°C (N = 19), 27°C ± 5°C (N = 26), 30°C ± 0°C (N = 25) and 30°C ± 5°C (N = 22). Sample sizes varied among treatments because individuals that had molted into adults were removed from the experiment. In the variable thermal treatments (*i*.*e*., ± 5°C, accuracy: ± 1°C and precision: 0.2°C), variability was achieved during the light hours (*i*.*e*., 12L). The temperature started to increase linearly at 7:00 h, reaching its maximum at 8:00 h, then stayed constant, and began to decrease at 19:00 h, reaching its minimum at 20:00 h (for more details, see Figure A of S1 Text). The heating/cooling rate between the minimum and maximum temperatures was 0.16°C/min. Thermal acclimation lasted five weeks and individuals were fed once during the acclimation using the same protocol as in the initial section and two weeks previous to TPCs measurements.

Low temperatures in winter had been proposed as the most limiting factor in population growth of *T*. *infestans* [38, 43, 44]. In the central region of Chile, a temperature rise of 4°C to 5°C is projected in the worst case scenario, while the scenario B2 predicts an increment of 2°C to 4°C [45]. Currently, the minimum winter temperature in the regions close to wild *T*. *infestans* foci is 10.7°C. However, this species inhabits peridomestic habitats, which buffer temperature fluctuations [28], a further feature that needs to be considered. For instance, this micro-site temperature buffer can reach up to ± 5°C to ± 8°C, inside of peri-domestic structures or houses, respectively [28]. Therefore, the lowest temperature to which we acclimated individuals resembled winter temperatures in their microenvironment (*e*.*g*., a peri-domestic structure) plus 2°C, which is equivalent to a conservative climate change scenario. Individuals were acclimated to these low temperatures with and without the inclusion of thermal variability (*i*.*e*., 18°C ± 5°C and 18°C ± 0°C, respectively). Additionally, since the thermal optimum for growth in *T*. *infestans*, is easily reached inside of human houses [46], we also acclimated individuals to their optimal temperature, with and without thermal variability (*i*.*e*., 27°C ± 5°C and 27°C ± 0°C, respectively). Finally, we acclimated individuals to the regional, maximum mean summer temperature of 30°C, also with and without thermal variation (*i*.*e*., 30°C ± 5°C and 30°C ± 0°C, respectively).

### Locomotor performance

To quantify the effect of temperature on locomotor performance, we measured the walking speed of four to fifth-instar nymphs from different acclimation treatments, for a description of sample sizes and body weight per treatment see Table A in S1 Text. We quantified the individual’s walking speed by measuring the time required to walk a distance of 15 cm in a rectangular track of the following dimensions: 15 cm x 1.5 cm and vertical walls of 1 cm. Prior to the measurement of walking speed, each individual was kept for one hour at the test temperature, keeping the following temperature sequence: 8°C, 18°C, 28°C, 34°C, 38°C, 40°C, 42°C, and 43°C. Between tests, all animals remained in a room at 22°C ± 2°C for at least one hour to minimize the effects of acclimation. The walking speed measurements were made in the morning and individuals were exposed to three temperatures per day, following the temperature sequence described above, to avoid stress effects on walking speed after multiple walking trials. Additionally, using the aforementioned test temperature sequence, we avoided losses of individuals early during experimental trials, due to higher mortality after exposure to the highest temperatures; and were able to estimate complete TPCs for single individuals [47]. Besides, using this protocol we reduced the sample size per thermal treatment. Nevertheless, to discard a learning effect during assays, we measured the walking speed in a different set of individuals following the same sequence but with the same exposing temperature as control (Figure B in S1 Text). We did not find any significant difference between measurements (Figure B and Tables B, C and D in S1 Text), thus, we can dismiss a possible learning effect. Before individuals were exposed to the eight test temperatures, they were weighed on the calibration scale BOECO (Model BAS 31plus), to assess total body mass (m_b_).

### Data analyses

Thermal performance curves vary with acclimation and their shape could take a wide range of phenological curves [48, 49]. Here, we studied how an individual’s TPC, and the individual’s thermal tolerance, varies with thermal acclimation, which essentially captures putative responses to climate change (*i*.*e*., an increase in average temperature and in its variance). Therefore, to analyze acclimation effects on TPCs, we used generalized additive models (GAMs) since this method does not make *a priori* assumptions about the shape of the relationship between the parameters: temperature and performance. This is key to analyze the effect of temperature (Tmp) on walking speed, and how the shape of the TPC changes with thermal acclimation (T). Moreover, the main difference between GAMs and linear models is that the linear functions of the variables in GAMs are replaced by unknown smooth functions (*e*.*g*., s, smooth; te, full tensor and ti, tensor product interaction), giving additional flexibility to the modelling process [50]. Considering that walking speed was measured for the eight exposure temperatures for each individual, we nested models by individual, according to Wood [50]. We also included as linear predictor variable the effect of thermal acclimation (T) and the m_b_. Moreover, our models allowed for the variation of curve shapes across thermal treatments (T) to be able to assay TPC changes with acclimation. The complexity of the curve (*i*.*e*., the number of degrees of freedom) and the smoothing terms were determined by penalized regression splines and generalized cross-validation (GCV; [50–53]) to avoid overfitting [50]. Also, we permitted shrinkage of the smoothers, allowing the addition of an extra penalty in the model: if the penalty is high enough, it will shrink all smoothing coefficients to zero. In other words, a penalty above a certain threshold, will remove the effect of the variable from the model, a procedure similar to the stepwise variable selection [54]. Model selection was done using the second-order Akaike Information Criterion (*i*.*e*., ΔAICc < 2; [55]) and the log likelihood ratio test, which is especially powerful testing differences between nested models, as ours [54]. After model selection, we performed a posterior simulation with the best model’s parameters to estimate the optimum temperature (T_o_), maximum locomotor performance (V_max_), and lower and upper thermal limits (LTL and UTL, respectively). We define LTL and UTL as the temperature where ten percent of the maximum locomotor performance (per each treatment) was achieved by the individual. This means that for thermal limits we estimated the ten percent of V_max_ for each treatment. We did this simulating 1000 coefficient vectors of the best model using the MASS package in R to estimate the 95% confidence intervals for the curve’s parameters on the temperature axis (*i*.*e*., T_o_, LTL, and UTL). For V_max_, we displayed standard deviations estimates using the *predict* function from the mvcg package, also in R [53].

## Results

**Fig 1:**
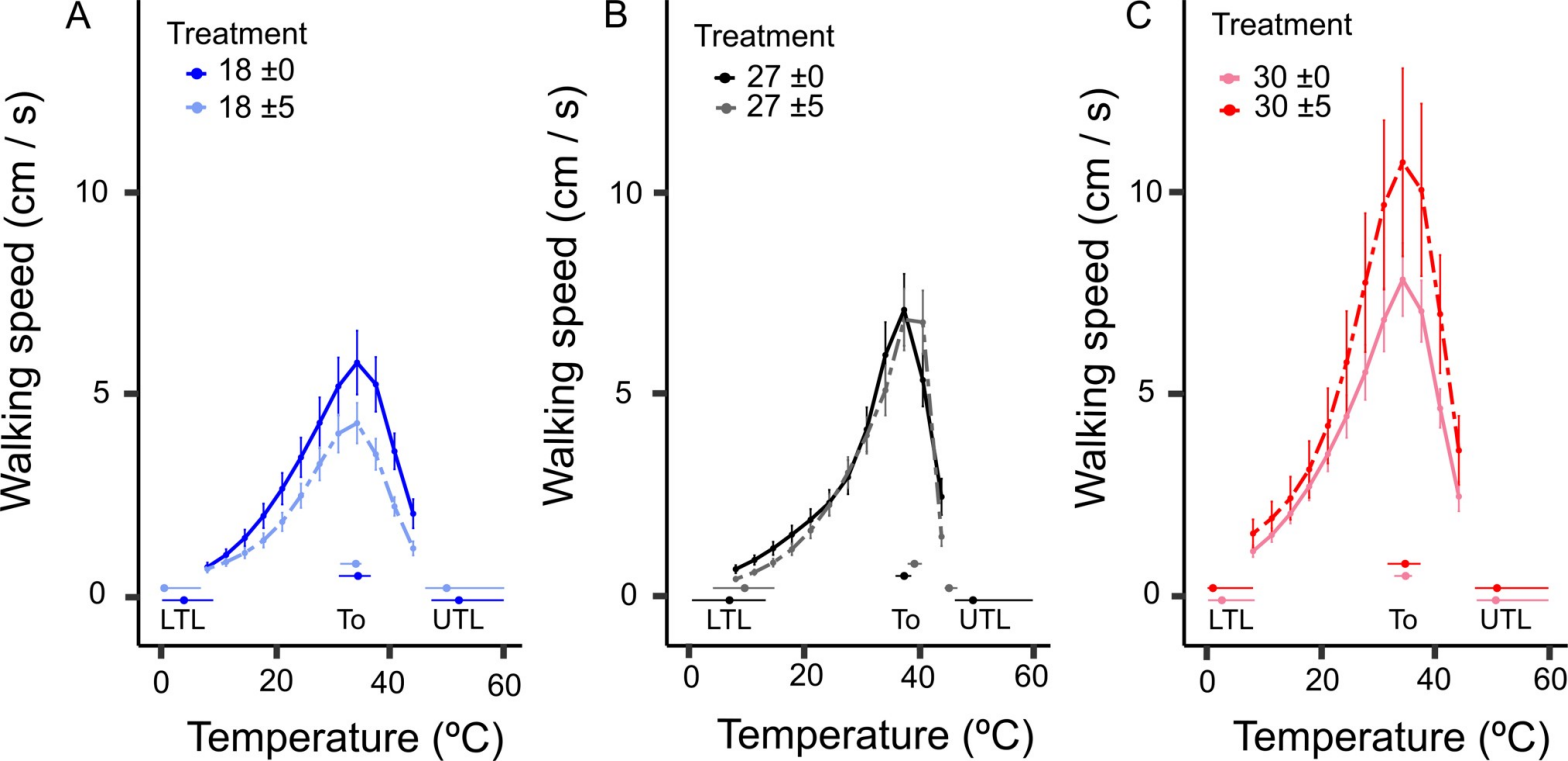
Thermal performance curves (TPCs) of *Triatoma infestans* acclimated to six thermal treatments, with and without daily thermal variability (±5°C and ± 0°C, respectively). A) TPCs for individuals acclimated to 18°C ± 0°C and 18°C ± 5°C (in blue); B) TPCs for individuals acclimated to 27°C ± 0°C and 27°C ± 5°C (in black), and C) TPCs for individuals acclimated to 30°C ± 0°C and 30°C ± 5°C (in red). Note that body mass was a significant variable affecting locomotor performance, and also interacted with thermal acclimation. Therefore, the predicted locomotor performance for an average individual’s body mass per treatment, is shown. Error bars show the standard error of the mean for walking speed. The optimum temperature (T_o_) was estimated as the temperature were the maximum locomotor performance (V_max_) was achieved per treatment (indicated with a colored dot, along the x-axis, according to the treatment). The lower (LTL) and upper thermal limits (UTL) were estimated for those temperatures were 10% of the maximum locomotor performance (V_max_) per treatment was achieved (LTL and UTL; also given with color-coded dots, for each treatment, on the x-axis). The 95% confidence interval for T_o_, LTL, and UTL were estimated using a GAM posterior simulation, and are indicated with colored horizontal bars, along the x-axis of each treatment, for each of the aforementioned values.

The non-linear relationship between temperature and performance changed with thermal acclimation. Further, the body mass (m_b_) also had an important role in modifying *T*. *infestans’* performance. Among the candidate GAM models, the best fit was achieved for the model including all predictor variables with a spline between walking speed and temperature (Tmp), per thermal acclimation treatment; *i*.*e*., s(Tmp, by = T), a linear m_b_, and the thermal acclimation treatment (T), plus the interaction between m_b_ and T (Table 1). The smooth term, s(Tmp, by = T), implied that the performance varied among thermal treatments (T), changing TPC shapes with treatment. As previously mentioned, we also controlled for the factor individual, due to our nested data structure, including a spline between walking speed and temperature, *i*.*e*., s(ID) (Table 1). Altogether, given the best competing model (Table 1), the TPC shapes changed with thermal acclimation (Table 1 and Fig 1). This model explained 62% of the variance evidencing a good fit for performance, in *T*. *infestans*.

**Table 1:** Model selection for GAM models of locomotor performance in *Triatoma infestans* acclimated to six thermal treatments that differed in thermal mean and in thermal variability.

Models for walking speed (WS)	df	AICc	weights	R^2^	F	P value
**s(Tmp, by = T) + s(ID) + m**_**b**_ **+ T + T x m**_**b**_	895.16	3969.74	0.236	0.625		
**s(Tmp) + s(ID) + m**_**b**_ **+ T + T x m**_**b**_	922.53	4084.38	0.000	0.591	6.50	< 0.001
**s(Tmp, by = T) + s(ID) + m**_**b**_ **+ T**	894.60	3971.19	0.214	0.624	5.36	< 0.05
**s(Tmp, by = T) + s(ID) + T**	893.95	3971.68	0.197	0.624	5.31	0.053
**s(Tmp, by = T) + s(ID) + m**_**b**_	893.73	3972.85	0.217	0.625	6.49	< 0.05
**s(Tmp, by = T) + s(ID)**	890.47	3974.58	0.136	0.625	5.32	< 0.05
**s(Tmp, by = T)**	1028.72	4593.29	0.000	0.274	9.10	< 0.001
**s(ID)**	933.06	5011.41	0.000	0.213	44.9	< 0.001

WS = walking speed, m_b_ = body mass, T = thermal treatment, Tmp = test temperature, ID = individual identification, s = smooth terms for temperature and individual (*i*.*e*., s(Tmp) and s(ID), respectively).

At 18°C, we found that animals acclimated to temperature variability decreased maximum performances (V_max_; Table 2 and Fig 1A). Regarding the thermal limits, the lower thermal limit (LTL) was similar for all thermal treatments (Table 2 and Fig 1). Despite that LTL between individuals acclimated to 18°C ± 0°C and 18°C ± 5°C did not differ, the performance at these low temperatures, below the temperature optimum (T_o_), were different, especially close to 20°C (Fig 1). Individuals acclimated to variable low temperatures (18°C ± 5°C) perform worse than individuals acclimated to constant low temperatures (18°C ± 0°C). The latter also performed worse at T_o_, reaching lower V_max_ values, and they had lower performance along the entire curve (Table 2 and Fig 1A). Nevertheless, the upper thermal limit (UTL) was not different between both thermal treatments at 18°C (Table 2).

**Table 2:** Parameters of the thermal performance curves (TPCs) of different thermal treatments, estimated from the best GAM model of walking speed in *Triatoma infestans*.

Thermal treatment	V_max_ (± sd)	LTL (95% Conf. interval)	UTL (95% Conf. interval)	T_o_ (95% Conf. interval)
**18°C ± 0°C**	5.780^a^ ±0.79	3.901^a^ (0.074–9.031)	52.005^a^ (47.183–59.847)	34.354^a,b,c,d^ (30.989–40.559)
**18°C ± 5°C**	4.285^b^ ±0.50	0.453^a^ (0.293–6.867)	49.848^a,b^ (46.104–59.920)	33.972^a^ (31.229–34.946)
**27°C ± 0°C**	7.073^a^ ±0.89	6.907^a^ (0.341–13.245)	49.508^a,b^ (46.322–59.959)	37.467^b*^ (35.948–38.711)
**27°C ± 5°C**	5.780^a^ ±0.69	9.564^a^ (4.008–14.774)	45.317^b*^ (44.535–46.778)	39.258^c*^ (38.073–40.559)
**30°C ± 0°C**	7.850^c^ ±0.91	2.552^a^ (0.135–8.282)	50.467^a^ (47.119–59.681)	34.693^a^ (32.698–35.815)
**30°C ± 5°C**	8.579^c^ ±1.06	0.986^a^ (0.039–8.005)	50.178^a^ (46.810–59.678)	34.622^a,b^ (31.525–37.301)

The confidence intervals of lower thermal and upper thermal limits (LTL and UTL respectively), and optimal temperature (T_o_) were estimated using a GAM posterior simulation. The letters in superscript show thermal treatment differences in the TPCs parameters and letters in superscript with an additional asterisk show marginal differences.

At 27°C, which is the optimum rearing temperature for this species, a thermal variability decreased V_max_, but the decrease was not significant (Table 2). Despite that the lower thermal limit (LTL) was similar between 27°C ± 0°C and 27°C ± 5°C, the UTLs slightly overlapped, for which the UTL was lower for the treatment with thermal variability compared to that of the treatment at a constant temperature of 27°C (Table 2). This increment in V_max_ and the associated decrease in UTL in individuals acclimated to thermal variability may be evidence of a trade-off between V_max_ and UTL. Regarding T_o_, there was a positive displacement among the individuals at 27°C ± 5°C, and the confidence intervals were once more tightly linked (Table 2).

At high temperatures (*i*.*e*., 30°C), the thermal variability also increased variation in V_max_. Nevertheless, there was no significant differences in V_max_ between both high temperature treatments, with or without thermal variations (Fig 1C). However, at high temperatures, V_max_ was greater than in both treatments at 27°C (*i*.*e*., with and without thermal variations; Fig 1B and 1C). Moreover, T_o_ did not show significant differences between thermal acclimation for 30°C ± 0°C and 30°C ± 5°C; and while LTL and V_max_ did not show differences with and without thermal variation at 30°C, the T_o_ decreased with an increase in mean and thermal variation by nearly two degrees Celsius (Fig 1C); but the UTL remained similar in both thermal acclimation treatments (Fig 1C).

**Fig 2:**
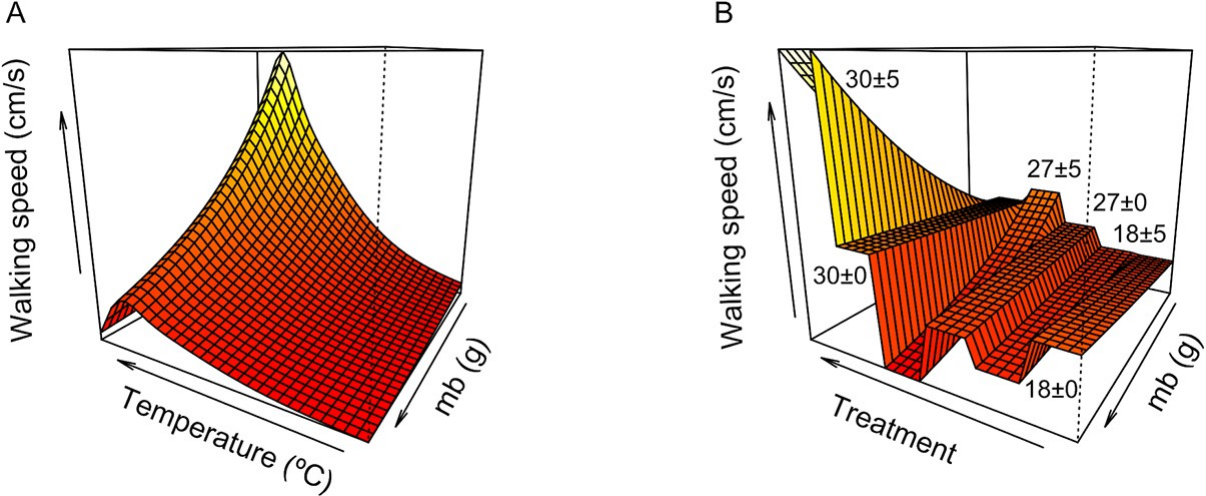
Body mass (m_b_) effects on performance curves and their interaction with temperature and thermal treatment in *Triatoma infestans*. A) Relationship between temperature and m_b_ and its effect on walking speed: at increased m_b_, performance decreased. B) Relationship between thermal treatment and m_b_: most thermal treatments exhibited a negative effect of m_b_ on performance (*i*.*e*., 18°C ± 0°C, 18°C ± 5°C, 27°C ± 0°C, 27°C ± 5°C, and 30°C ± 0°C), with a greater slope in treatments with thermal variation (*i*.*e*., ±5°C). However, for 30°C ± 5°C, the relationship was positive, with bigger individuals performing better than smaller ones.

In addition to the effects of acclimation on performance, we also found a negative effect of m_b_ on performance, with smaller kissing bugs performing better than larger ones (Fig 2). Despite that in almost all the acclimation treatments this negative relationship between m_b_ and performances is evident (*i*.*e*., 18°C ± 5°C, 18°C ± 0°C, 27°C ± 0°C, 27°C ± 5°C, and 30°C ± 0°C), this relationship was positive for individuals acclimated to 30°C ± 5°C (Fig 2). Thus, only larger individuals acclimated at 30°C ± 5°C performed better than smaller ones (Fig 2).

## Discussion

Environmental variability in time and space imposes selective pressures on organisms, driving adaptation (or not) to vary thermal environments. Adaptation is simultaneously a function of the time pattern of environmental changes and the phenotypic tolerance of each phenotype [16]. Theoretically, animals that inhabit variable environments are expected to exhibit plastic strategies that may enable them to survive a broad range of temperatures [47].

Within this context, we found a strong effect of thermal variability on the thermal performance of *Triatoma infestans* individuals (Fig 1A, 1B and 1C). In fact, the thermal performance curves (TPCs) changed appreciably with thermal acclimation in both, thermal constant and thermal variable treatments. Moreover, these results showed that despite that lower (LTL) and upper thermal limits (UTL) did not change significantly, the non-linear relationship between performance and temperature did change (Fig 1). We may argue that the study of thermal limits is useful as a first approximation to understand the tolerance of *T*. *infestans* to climatic variable conditions, but it was not sensible enough to capture changes along the species’ thermal niche [56].

The changes in the curves’ shape were evidenced with the significant cubic spline between walking speed and temperature, s(Temp, by = T), included in the best fitted model, as shown in Table 1. Regarding the thermal limits, our estimation of thermal limits was superior (*i*.*e*., 1°C to 2°C) to previous estimations for a critical thermal minimum (CT_min_) and a critical thermal maximum (CT_max_) [57]. Nevertheless, we estimated the LTL and the UTL as the temperature were 10% of the maximum performance (V_max_) was reached (see methodology), whereas the thermal limits in Belliard et al. [57] were estimated using standard protocols recording a complete loss of the individual’s motoric capacity [58]. Moreover, we acclimated individuals during five weeks with one feeding event, while the thermal acclimation of the aforementioned study was shorter, lasting only one week [57]. Thus, our estimations were slightly different from previous estimations for thermal limits for this species.

Theoretical and empirical approaches indicate that rapid environmental climatic changes impact both, the mean temperatures of local environments and the magnitude of geographical, diel, and seasonal variations in temperature [22]. Our results showed that environmental variation had negative effects on performance at a low temperature (Fig 1A). In fact, thermal variation decreased V_max_ and the individuals’ performance along the entire sequence of temperatures of exposure when the individuals were acclimated to a low temperature (18°C ± 5°C). Despite that the curve’s shape changed noticeably in individuals acclimated to 18°C ± 0°C, in comparison to both thermal treatments at 27°C, there was no significant decrease of V_max_ (Fig 1A and 1B and Table 2). These results are relevant, since low environmental temperatures, such as those in winter, have been suggested as the principal factor affecting densities of wild populations of *T*. *infestans* [43]. In fact, a study on thermal tolerances and geographic distributions of seven triatomine species, including *T*. *infestans*, evidenced that species from subtropical and temperate regions, exhibit higher CT_min_ values than the minimum temperature of the coldest month [59]. Our results are evidence that changes in performance are negligible at constant, low temperatures, while low and variable environmental temperatures caused an important reduction in performance (Fig 1A). However, at a high temperature, of 30°C, thermal variability had the opposite effect of that observed at a low thermal acclimation (Fig 1A and 1C). In other words, thermal variability increased performance at 30°C, particularly at temperatures above the optimum temperature (T_o_, see Fig 1C), but V_max_ was not different between both 30°C thermal treatments, with or without thermal variability (Table 2). Summarizing, thermal variability: (a) had a strong negative effect at a low temperature (*i*.*e*., at 18°C), decreasing performance along the thermal niche of *T*. *infestans*; (b) displaced T_o_ and decrease ULT marginally at 27°C (Fig 1B); and (c) had a slightly positive effect on performance at high temperatures (*i*.*e*., at 30°C, Fig 1C).

The evidence we have of thermal effects on locomotor performance in *T*. *infestans* acclimated to thermal treatments with and without variability, may imply dependent fitness response to climate change. However, it is important to point out that while locomotor TPCs are a well-established proxy for fitness (due to their link to reproduction and survival [60–63]), TPCs also vary in shape and in performance breath across organization levels [64]. This means that when estimating TPCs at the physiological level (*e*.*g*., metabolism and thermal limits), the performance breath is wider than among estimations at the population level (*e*.*g*., population growth). Therefore, the shape of TPCs of other traits, such as survival and fecundity, could be slightly different [65] and should be targeted by future studies in *T*. *infestans*.

The consequences of thermal variability for the performance of *T*. *infestans’* individuals under different climatic scenarios are more than complex, making it very difficult to make projections at a population level. We acclimated individuals at a low temperature representative of a peri-domestic site in winter, with an increased mean temperature (by 2°C), plus a thermal variability of five degrees Celsius (18°C ± 5°C; [43, 44]). Under this scenario, climate change will affect the performance of individuals during winter, negatively, and probably they will be less active early in spring due to winter acclimation, especially around 20°C and 25°C (Fig 1A). Nevertheless, an increase of 2°C around the mean of winter temperatures (represented in our 18°C ± 0°C treatment) did not display a substantial difference in *T*. *infestans’* performance compared to the performance at its optimum rearing temperature (*i*.*e*., 27°C), which can be easily reached in a domestic site. Along these lines, theoretical models [18, 66] predict that changes in mean temperature and in its variability close to the inflexion point of TPCs could produce mixed effects [18], as observed in this study. For example, the theoretical model proposed by Estay et al. [18] predicts that an increase of mean temperature around the inflexion point of TPCs will increase performance, whereas an increase of thermal variability will produce a non-linear negative effect [18], such as we observed in our results (Fig 1A). It is plausible that temperatures around 18°C, as those currently present in peri-domestic microsites of *T*. *infestans* during winter, represent the inflexion point of the curve for the population. Thus, we argue that low winter temperatures are probably a limiting factor in populations of *T*. *infestans*, but not for so much range of temperatures. Another relevant factor is the blood supply at the microsites. Notice that our individuals of *T*. *infestans* were fed *ad libitum* once during the five weeks of acclimation. Thus, we cannot argue that those feeding conditions represent natural conditions, which are likely to be more limited. Consequently, it is necessary to include body mass (m_b_) effects on performance to fully understand population responses to temperature changes.

Indeed, m_b_ had a negative effect on performance, in *T*. *infestans* (Fig 2A and 2B); and we only observed an increment in performance with m_b_ at the 30°C ± 5°C thermal treatment (Fig 2A and 2B). This may indicate that at a high and variable temperature, if the environment offers plenty of food sources, *T*. *infestans* may perform better than if only the temperature effect is considered. Along this thought, the sylvatic foci in Chile unfortunately have plenty of blood sources available since the marsupial *Thylamys elegans* and rabbits (*e*.*g*., *Oryctolagus cunniculus*) nest in close vicinity [33].

We did not explore the mechanisms explaining the m_b_ effects on performance, for which they remain uncertain. Nevertheless, feeding on blood, an energetic low-quality food, may induce the intake of copious volumes to get all the energy necessary to live. Hence, a trade-off becomes evident, where individuals full of blood (*i*.*e*., bigger individuals), may on the one hand move slower (because they are heavier) than smaller ones, which on the other hand need to be faster in order to find food and forage. Nevertheless, at high and variable temperatures (*i*.*e*., 30°C ± 5°C), the metabolism and physiological functions are higher, and those individuals with energetic reserves (*i*.*e*., bigger individuals) performed better. That may be the reason why we only observed a positive effect of m_b_ on performance at 30°C ± 5°C, and not a negative effect of a heavier body struggling to move faster. However, this aspect remains to be studied in more detail. A previous study in a related triatomine species, *R*. *prolixus*, evidenced thermal variability costs, with males exhibiting lower m_b_ and females lower fecundity at 24°C ± 7°C, than those individuals reared at constant temperature (*i*.*e*., 24°C) [67]. To our knowledge, apart from our work, there are no similar studies in *T*. *infestans* that could help elucidate m_b_ effects on performance.

Regarding the TPC parameters, we already discussed the thermal effects on V_max_ and on thermal limits. Nevertheless, the thermal optimum (T_o_) also changed with thermal acclimation. Moreover, T_o_ was greater than the optimum rearing temperature (*i*.*e*., 27°C to 28°C; [46]) for all individuals, regardless of their thermal acclimation treatment (Table 2). The lowest T_o_ value for walking speed was 33.97°C in individuals acclimated to 18°C ± 5°C, and was different from T_o_ values estimated for the 27°C treatments; but not from those estimated for both 30°C treatments and the 18°C ± 0°C treatment (Table 2). This means that under thermal variability at a low temperature, the largest decrease in T_o_ was recorded. Thermal variability at 27°C, marginally increased the T_o_ value (*i*.*e*., displacing it to the right; Fig 1), while the UTL was decreased (*i*.*e*., displacing it to the left; Fig 1B) compared to the 27°C ± 0°C thermal treatment. At 30°C, T_o_ values were not different between both acclimations, with and without thermal variability, and these T_o_ values were similar to those at a low temperature (Table 2). Thus, the highest T_o_ was found among individuals acclimated to 27°C ± 5°C, while a high temperature (*i*.*e*., 30°C) decreased thermal optima in this species.

Today, the Chagas disease treatment is limited to the early stages of infection and is highly effective. Despite the effectiveness of this treatment, there are many new cases of Chagas per year in South America because most of the populations at risk do not have access to timely treatment or even general medical care. The absence of vaccines or other effective preventive measures against Chagas infection, leaves vector population control as the most effective plan to prevent this disease [25]. In this context, our results revealed the relevance and complexity of the thermal response of *T*. *infestans*, the main vector of Chagas disease in southern South America, to thermal variation. This species, living in temperate places, seems to be extremely sensible to thermal variability at low temperatures, an observation that could help control the vector’s populations considering only climate change effects of the coldest months. However, at a constant, low temperature, this species performs as well as at its optimal temperature, and exhibits a wide plastic response to thermal variability, increasing its performance at a high temperature with thermal variability. This plastic response and a non-linear effect of thermal variability on the performance of *T*. *infestans* posits difficulties in predicting changes in the vector’s distribution under the rapid current climate change. Moreover, our results pose doubts about former optimistic projections on the distributions of Chagas’ vectors under climate change from studies which did not include thermal plasticity or the effects of thermal variability [68].

## Supporting information

S1 Text **Figure A S1 Text**. Temperature’s readings of climatic chambers were thermal treatments were performed. **A**) Temperature record during seven days of climatic chamber set it at 27±0°C. **B**) Temperature record during seven days of climatic chamber set it at 27±5°C. Pink line shows the set temperature values, blue line is climatic chamber’s temperature, light blue line is environment temperature outside the chamber and green line shows the control temperature (probe inside a glass vessel in the chamber). **Table A S1 Text.** Mean body mass (mb) per treatment and developmental stage (i.e., instar) before and after thermal acclimation (i.e., treatment). Note that TPCs were estimated at the end of acclimation and all individuals that molted to adult were removed from the experiment. **Figure B S1 Text.** Control experiment, individuals were exposed at the same temperature following the same procedure performed for TPC estimation (see Methodology). First, we exposed individuals at 8 consecutive measures (m8, m18, m38, m40, m42, m43, and m44) corresponding to the eight exposing temperatures for TPC estimation. We repeated this procedure for two temperatures (*i*.*e*., 18°C and 38°C, left and middle panel). Besides, we performed the same procedure, exposing individuals at 18°C, but these individuals were acclimated during two weeks under a reverted photoperiod of 12D:12L (right panel). Note this data is not corrected by body size. **Table B S1 Text**. Model summary for walking speed of individuals measured at 18°C in the control experiment. We did no find a significant effect of the consecutive measure on walking speed. **Table C S1 Text**. Model summary for walking speed of individuals measured at 38°C. We did no find a significant effect of the consecutive measure on walking speed. **Table D S1 Text**. Model summary for walking speed of individuals measured at 18°C with different photoperiod (R: reverted). Cm) consecutive measures, mb) body mass and photoperiod as categorical variable (normal and reverted).
(DOCX)
