# Supplementary material for: Thermal performance of the Chagas disease vector, Triatoma infestans, under thermal variability
Source: PLoS Negl Trop Dis. 2021 Feb 11;15(2):e0009148. doi: 10.1371/journal.pntd.0009148 (PMC7904210; doi:10.1371/journal.pntd.0009148)
Supplement: S1 Text — Figure A S1 Text. Temperature’s readings of climatic chambers were thermal treatments were performed. A) Temperature record during seven days of climatic chamber set it at 27±0°C. B) Temperature record during seven days of climatic chamber set it at 27±5°C. Pink line shows the set temperature values, blue line is climatic chamber’s temperature, light blue line is environment temperature outside the chamber and green line shows the control temperature (probe inside a glass vessel in the chamber). Table A S1 Text. Mean body mass (mb) per treatment and developmental stage (i.e., instar) before and after thermal acclimation (i.e., treatment). Note that TPCs were estimated at the end of acclimation and all individuals that molted to adult were removed from the experiment. Figure B S1 Text. Control experiment, individuals were exposed at the same temperature following the same procedure performed for TPC estimation (see Methodology). First, we exposed individuals at 8 consecutive measures (m8, m18, m38, m40, m42, m43, and m44) corresponding to the eight exposing temperatures for TPC estimation. We repeated this procedure for two temperatures (i.e., 18°C and 38°C, left and middle panel). Besides, we performed the same procedure, exposing individuals at 18°C, but these individuals were acclimated during two weeks under a reverted photoperiod of 12D:12L (right panel). Note this data is not corrected by body size. Table B S1 Text. Model summary for walking speed of individuals measured at 18°C in the control experiment. We did no find a significant effect of the consecutive measure on walking speed. Table C S1 Text. Model summary for walking speed of individuals measured at 38°C. We did no find a significant effect of the consecutive measure on walking speed. Table D S1 Text. Model summary for walking speed of individuals measured at 18°C with different photoperiod (R: reverted). Cm) consecutive measures, mb) body mass and photoperiod as categorical variable (normal and rever [file pntd.0009148.s001.docx]

**Supporting Information**

**Figure A**. Temperature’s readings of climatic chambers were thermal treatments were performed. **Upper panel**) Temperature record during seven days of climatic chamber set it at 27±0 ℃. **Lower panel**) Temperature record during seven days of climatic chamber set it at 27±5 ℃. Pink line shows the set temperature values, blue line is climatic chamber’s temperature, light blue line is environment temperature outside the chamber and green line shows the control temperature (probe inside a glass vessel in the chamber).


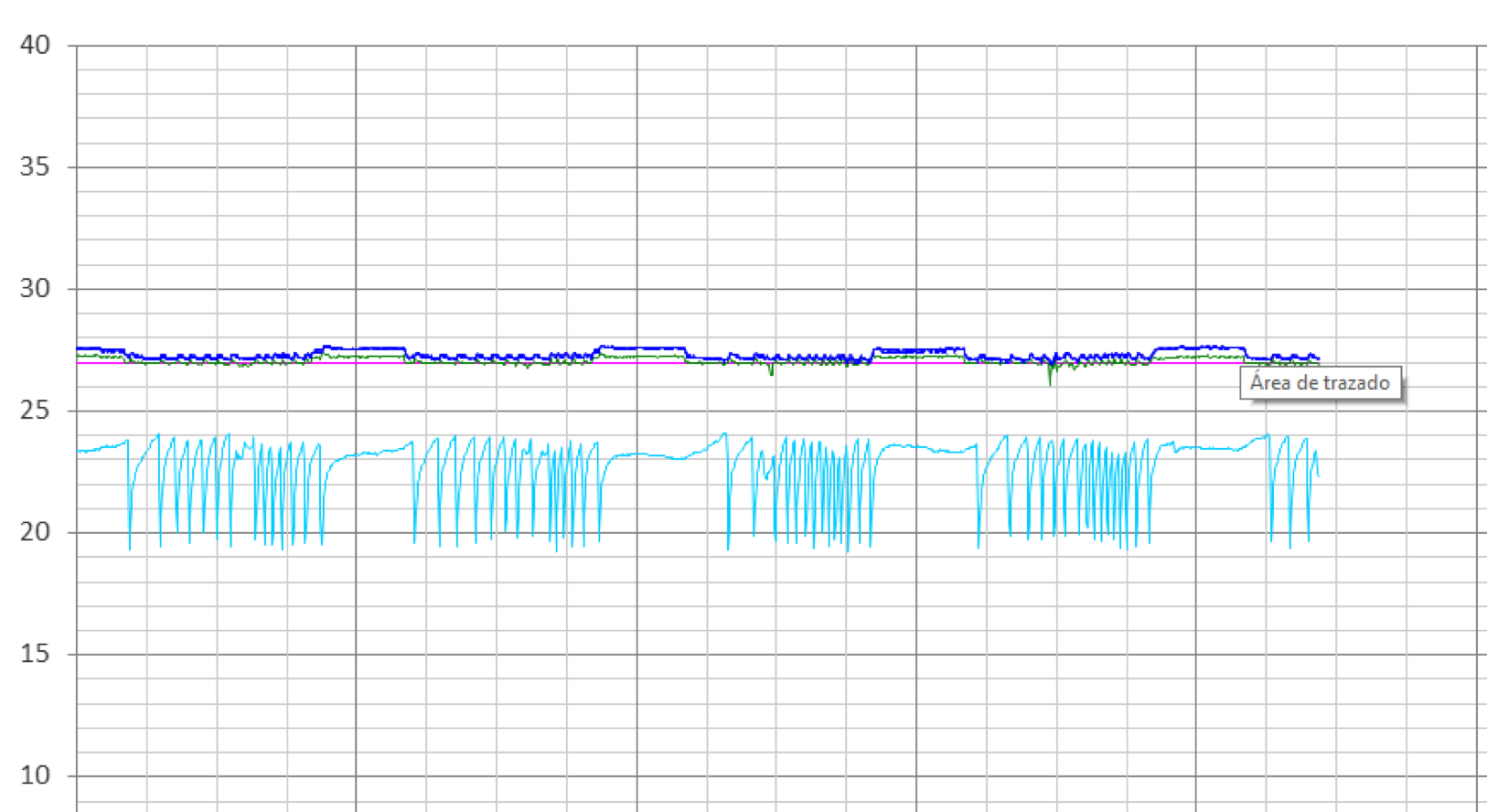


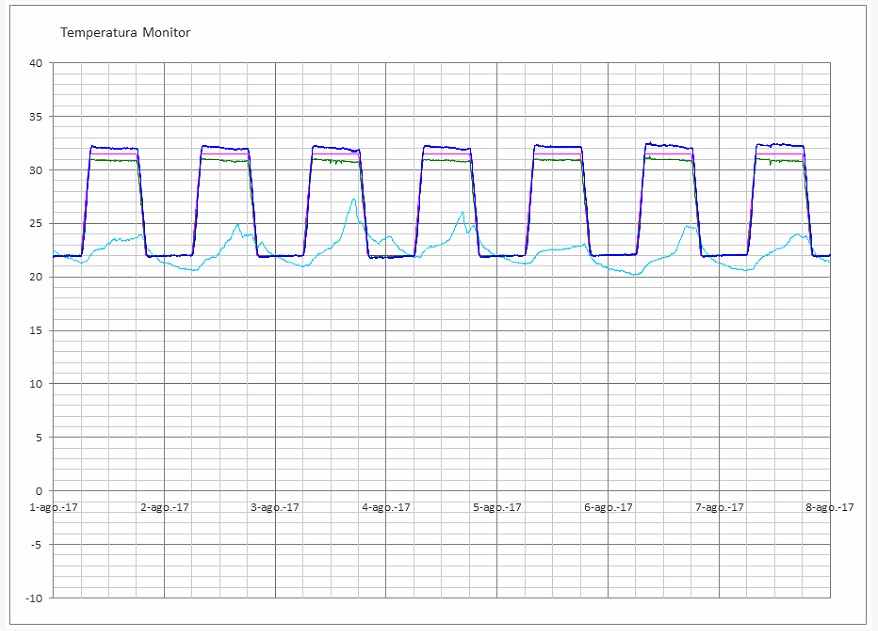


**Table A.** Mean body mass (mb) per treatment and developmental stage (i.e., instar) before and after thermal acclimation (i.e., treatment). Note that TPCs were estimated at the end of acclimation and all individuals that molted to adult were removed from the experiment.

|  | Before acclimation | | | | After acclimation | | | |  | |
| --- | --- | --- | --- | --- | --- | --- | --- | --- | --- | --- |
| Treatment | Mean  body mass | # 4th  instar | # 5th  instar | Mean  body mass | | # 4th instar | # 5th instar | Sample size | |  |
| 18±0 ℃ | 0,201 | 5 | 25 | 0,232 | |  | 17 | 17 | |  |
| 18±5 ℃ | 0,130 | 9 | 18 | 0,178 | | 3 | 21 | 24 | |  |
| 27±0 ℃ | 0,170 | 1 | 24 | 0,189 | |  | 19 | 19 | |  |
| 27±5 ℃ | 0,147 | 3 | 8 | 0,152 | | 1 | 25 | 26 | |  |
| 30±0 ℃ | 0,134 | 26 | 3 | 0,099 | |  | 25 | 25 | |  |
| 30±5 ℃ | 0,115 | 24 | 5 | 0,102 | | 1 | 21 | 22 | |  |

**Figure B.** Control experiment, individuals were exposed at the same temperature following the same procedure performed for TPC estimation (see Methodology). First, we exposed individuals at 8 consecutive measures (m8, m18, m38, m40, m42, m43, and m44) corresponding to the eight exposing temperatures for TPC estimation. We repeated this procedure for two temperatures (*i.e.,* 18 ℃ and 38 ℃, left and middle panel). Besides, we performed the same procedure, exposing individuals at 18 ℃, but these individuals were acclimated during two weeks under a reverted photoperiod of 12D:12L (right panel). Note this data is not corrected by body size.


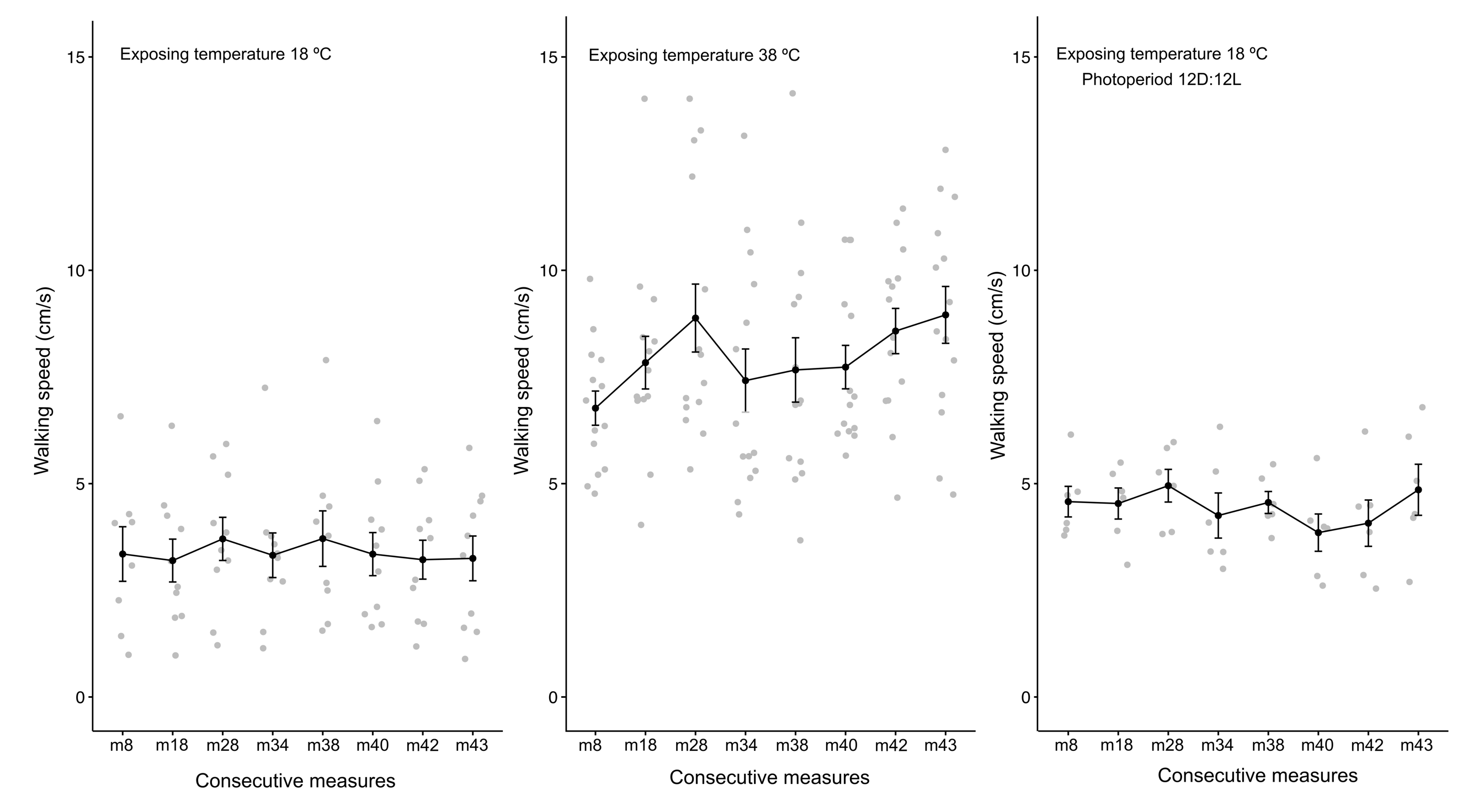


**Table B**. Model summary for walking speed of individuals measured at 18 ºC in the control experiment. We did no find a significant effect of the consecutive measure on walking speed.

| Variable | Estimate | s.e. | Residual  deviance | p value |
| --- | --- | --- | --- | --- |
| Intercept | 1.004 | 1.975 | 192.94 |  |
| Cm | 0.004 | 0.058 | 192.94 | 0.991 |
| mb | 32.158 | 25.702 | 185.37 | 0.083 |
| Cm x mb | -0.547 | 0.755 | 184.04 | 0.468 |

**Table C**. Model summary for walking speed of individuals measured at 38 ºC. We did no find a significant effect of the consecutive measure on walking speed.

| Variable | Estimate | s.e. | Residual  deviance | p value |
| --- | --- | --- | --- | --- |
| Intercept | 6.602 | 1.975 | 653.56 |  |
| Cm | 0.069 | 0.058 | 634.23 | 0.068 |
| mb | 3.713 | 25.702 | 631.29 | 0.477 |
| Cm x mb | -0.188 | 0.755 | 628.27 | 0.472 |

**Table D**. Model summary for walking speed of individuals measured at 18 ºC with different photoperiod (R: reverted). Cm) consecutive measures, mb) body mass and photoperiod as categorical variable (normal and reverted).

| Variable | Estimate | s.e. | Residual  deviance | p value |
| --- | --- | --- | --- | --- |
| Intercept | 3.119 | 0.476 | 280.80 |  |
| Cm | -0.004 | 0.011 | 280.46 | 0.680 |
| mb | 5.105 | 3.757 | 249.56 | < 0.001 |
| photoperiod (R) | 0.697 | 0.379 | 242.85 | 0.065 |
